# LPS O Antigen Plays a Key Role in Klebsiella pneumoniae Capsule Retention

**DOI:** 10.1128/spectrum.01517-21

**Published:** 2022-08-01

**Authors:** Shweta Singh, Jonathan J. Wilksch, Rhys A. Dunstan, Anna Mularski, Nancy Wang, Dianna Hocking, Leila Jebeli, Hanwei Cao, Abigail Clements, Adam W. J. Jenney, Trevor Lithgow, Richard A. Strugnell

**Affiliations:** a Department of Microbiology and Immunology, University of Melbourne at the Peter Doherty Institute for Infection and Immunity, Melbourne, Victoria, Australia; b Infection and Immunity Program, Biomedicine Discovery Institute and Department of Microbiology, Monash University, Clayton, Victoria, Australia; c Department of Life Sciences, Imperial College London, London, United Kingdom; Institut Pasteur

**Keywords:** encapsulation, LPS, *Klebsiella*, virulence, O antigen, capsule, retention

## Abstract

Despite the importance of encapsulation in bacterial pathogenesis, the biochemical mechanisms and forces that underpin retention of capsule by encapsulated bacteria are poorly understood. In Gram-negative bacteria, there may be interactions between lipopolysaccharide (LPS) core and capsule polymers, between capsule polymers with retained acyl carriers and the outer membrane, and in some bacteria, between the capsule polymers and Wzi, an outer membrane protein lectin. Our transposon studies in Klebsiella pneumoniae B5055 identified additional genes that, when insertionally inactivated, resulted in reduced encapsulation. Inactivation of the gene *waaL*, which encodes the ligase responsible for attaching the repeated O antigen of LPS to the LPS core, resulted in a significant reduction in capsule retention, measured by atomic force microscopy. This reduction in encapsulation was associated with increased sensitivity to human serum and decreased virulence in a murine model of respiratory infection and, paradoxically, with increased biofilm formation. The capsule in the WaaL mutant was physically smaller than that of the Wzi mutant of K. pneumoniae B5055. These results suggest that interactions between surface carbohydrate polymers may enhance encapsulation, a key phenotype in bacterial virulence, and provide another target for the development of antimicrobials that may avoid resistance issues associated with growth inhibition.

**IMPORTANCE** Bacterial capsules, typically comprised of complex sugars, enable pathogens to avoid key host responses to infection, including phagocytosis. These capsules are synthesized within the bacteria, exported through the outer envelope, and then secured to the external surface of the organism by a force or forces that are incompletely described. This study shows that in the important hospital pathogen Klebsiella pneumoniae, the polysaccharide capsule is retained by interactions with other surface sugars, especially the repeated sugar molecule of the LPS molecule in Gram-negative bacteria known as “O antigen.” This O antigen is joined to the LPS molecule by ligation, and loss of the enzyme responsible for ligation, a protein called WaaL, results in reduced encapsulation. Since capsules are essential to the virulence of many pathogens, WaaL might provide a target for new antimicrobial development, critical to the control of pathogens like K. pneumoniae that have become highly drug resistant.

## INTRODUCTION

Klebsiella pneumoniae is a primary and opportunistic pathogen that causes community-acquired and nosocomial infections (1). The treatment of *K. pneumoniae* infections has become increasingly problematic, with a rise in extended-spectrum β-lactamase (ESBL) resistance (2), an increase that has been correlated with antibiotic misuse (3). The resistance profile of K. pneumoniae extended further when it acquired the metallo-β-lactamase NDM-1 (New Delhi metallo-β-lactamase-1) (4, 5), which when combined with development of colistin resistance (6) severely limits practical treatment options. Thus, there is a pressing need to develop alternate means of controlling Klebsiella species infections, such as vaccine-mediated prophylaxis and antibody and bacteriophage therapies (7, 8).

The global phylogeny of K. pneumoniae is evolving (9–11), but pathogenic members of the K. pneumoniae species complex (KpSC) are characterized by the expression of a polysaccharide capsule that plays a vital role in the virulence of the bacterium (12); more than 80 capsular types have been identified (13, 14). KpSC capsular polysaccharides (CPSs) are immunogenic and nontoxic and have been used as immunogens in experimental human vaccines (15). A 24-valent vaccine was formulated by Cryz et al. as early as 1984 (16), but while there may be an increased frequency of K1 and K2 serotypes in clinical isolates (17, 18), and the K2 capsule is often found on hypervirulent strains (19), the presence of numerous capsular types within hospital settings (20, 21) has limited commercial development of a KpSC vaccine. Our attention has been focused instead on alternate means of controlling KpSC infections by blocking conserved mechanisms of capsule regulation, biosynthesis, secretion, and retention.

Considerable advances have been made toward understanding the biosynthesis and secretion of capsular polysaccharide in KpSC. The structural genes for capsular polysaccharide synthesis, designated *cps* genes, are located in an operon adjacent to the lipopolysaccharide (LPS) *rfb* locus (22, 23), situated between *galF* and *gnd* (24). Genes outside the capsule operon also play a role in capsule biogenesis (25–27), but capsule retention processes remain incompletely resolved. In some capsule types, there may be covalent linkage of the CPS through a conserved anchor to the cell surface (28). Noncovalent interactions between capsule polysaccharide and LPS core (27, 29) and capsule and the membrane-associated beta-barrel protein Wzi (30) have also been reported. While the capsule is protective, there are biological scenarios where reduced encapsulation may be advantageous: for example, nonencapsulated strains show increased binding to biotic and abiotic surfaces (20, 31), and reduction of encapsulation could facilitate biofilm formation. Similarly, since capsule can serve as a receptor for bacteriophage, regulation of capsule retention could provide for resistance to phage attack. The purpose of this study was to identify genes that are located outside the KpSC capsule synthesis locus that are involved in capsule biogenesis and retention. The study used transposon mutagenesis coupled with atomic force microscopy (AFM), biochemical assays, biofilm assays, and phage sensitivity combined with cell and animal biology to identify a role of the LPS-capsule interaction in bacterial virulence.

## RESULTS

### The capsule of K. pneumoniae B5055.

**FIG 1:**
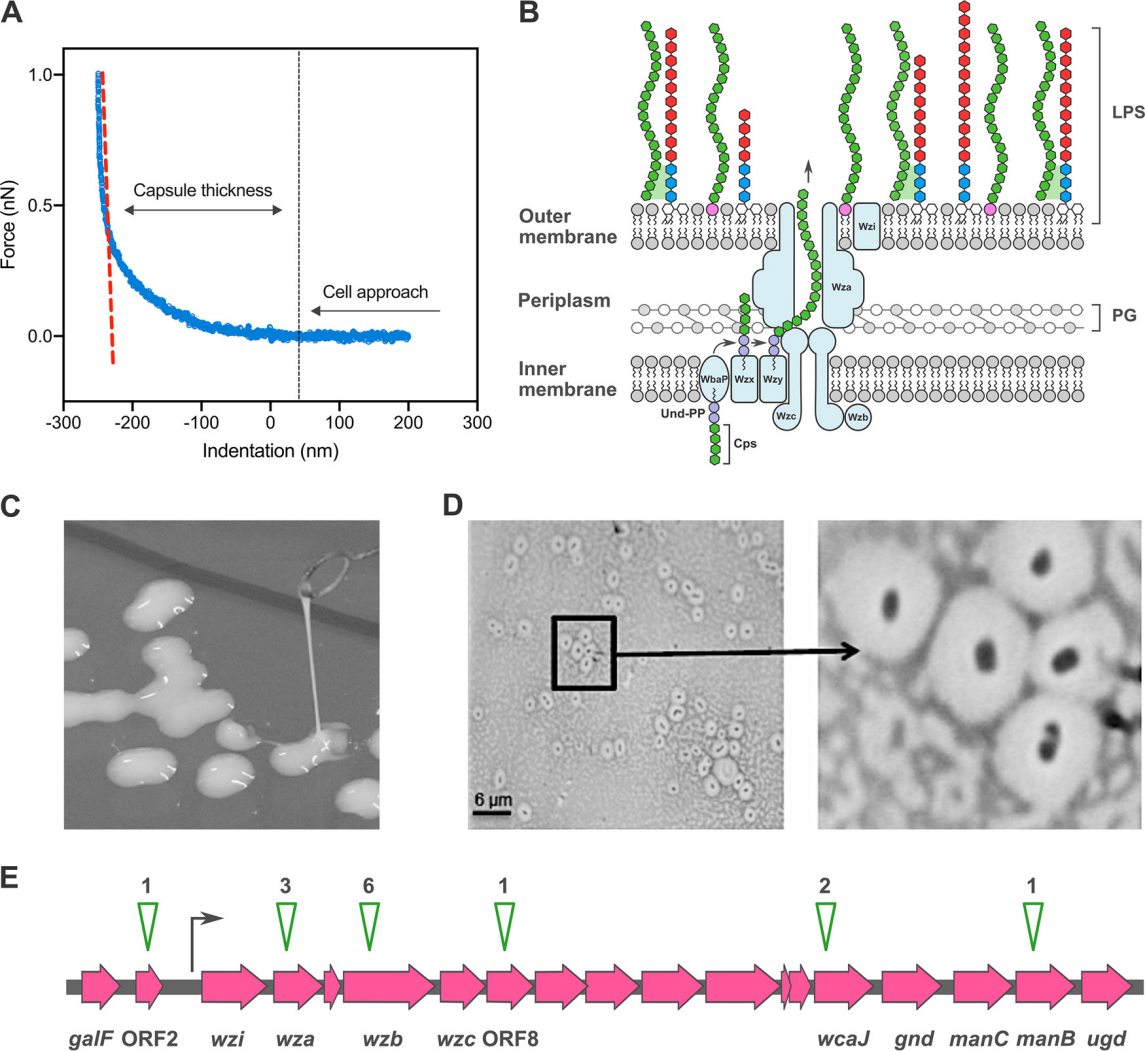
Capsule of Klebsiella pneumoniae B5055. (A) Measurements of single B5055 cells were made by atomic force microscopy to calculate capsule thickness. The dashed red line represents the fit of the curve to Hooke’s law, and blue circles represent individual measurements. (B) Representation of the capsule secretion machinery in K. pneumoniae, based on references 84 and 85. The secretion pore, composed of Wza, Wzb, and Wzc subunits, spans the inner membrane (IM) and outer membrane (OM). The beta-barrel outer membrane protein Wzi is depicted in proximity to the pore. For scale, the distance from the inside surface of the outer membrane to the peptidoglycan layer (PG) is approximately 10 nm, compared with the capsule thickness, which is >250 nm. (C) Colonies of K. pneumoniae B5055 grown on LB agar can be drawn into strings through contact with a bacteriological loop. These string lengths (i.e., at the point of breakage) were measured directly using a ruler. (D) Maneval stain of the encapsulated isolate B5055. (E) Eleven insertion mutants were identified within the *cps* gene cluster, predominantly within the *wza* and *wzb* genes that encode the capsule secretion pore subunits Wza and Wzb. The insertions within the CPS cluster identified in the capsule-deficient screen are denoted by green arrowheads, with the number of independent insertions shown above each arrowhead.

Klebsiella pneumoniae B5055 strain exhibits a mucoid phenotype, and measurements using atomic force microscopy (AFM) suggest a capsule thickness of 300 to 400 nm in solution surrounds the bacterial cells (Fig. 1A; see Table S1 in the supplemental material). The structural genes that encode the CPS secretion pore (*wza*, *wzb*, and *wzc*) (Fig. 1B) are located in a *cps* operon that also includes *wzi*. The gene *wzi* encodes a beta-barrel outer membrane protein, Wzi, that may be involved in capsule retention (30). The colonies of B5055 form a viscous “string” when contacted by a bacteriological loop (Fig. 1C), and a reduction or absence in string formation can be used to identify bacterial mutants with potentially reduced encapsulation.

A mini-Tn*5* transposon described by Lorenzo et al. (32) was used for mutagenesis of the K. pneumoniae mouse-virulent strain B5055 (33). A total of 8,400 colonies were screened for capsular defects, and 53 kanamycin-resistant colonies (KpSC01 to KpSC53) were selected based on a nonmucoid colony appearance, altered string test, and a negative or reduced Maneval’s stain. The two workflows used are shown in Fig. S1A; both yielded similar results. The observed reduction in string length and Maneval staining in the transposon mutants could reflect one or more of three phenotypes resulting from the mutagenesis: (i) a reduction in capsule polysaccharide synthesis, (ii) a reduction in capsule polysaccharide secretion, and/or (iii) a reduction in capsule polysaccharide retention. Further assays were conducted to separate these phenotypes for selected mutants.

The average of string test results for each mutant is shown in Fig. S1B. The B5055 Δ*wzb-c* strain carries a deletion of *wzb-wzc* and therefore lacks the capsule translocon (31) and served as the negative control for these studies. Forty-nine mutants were identified with significant (i.e., >50%) reduction in string length (Fig. S1B) compared with B5055 and for encapsulation by Maneval staining (Fig. 1D). Fourteen mutants had disruptions in the *cps* locus (Fig. 1E; Table S2). Capsule-deficient mutants were identified with transposon insertions within *wza*, *wzc*, *wcaJ*, and *manB*, open reading frame 2 (ORF2) (coding for a putative acid phosphatase), ORF8 (coding for a putative glycosyltransferase), and the promoter region of the CPS synthesis gene cluster (24). All the mutants that tested positive for a capsular defect in primary screening were examined for uronic acid, a component of the K2 CPS, in pelleted bacteria to estimate the quantity of CPS produced, secreted, and retained on the bacterial surface. Insertion mutant KpSC31, which carried a transposon insertion within the lipid A biosynthesis gene *msbB*, produced a longer string than B5055 (Fig. S1B), but a normal level of Maneval staining. The uronic acid associated with the pelleted KpSC31 was similar to the levels found in the parent strain, K. pneumoniae B5055 (see Fig. S2 below).

The percentage of uronic acid in K. pneumoniae B5055 is 22% by weight of capsular polysaccharide (34). Low levels of uronic acid are also present in the lipopolysaccharide (LPS) as galacturonic acid (GalA) (29). In order to establish the appropriate phase of growth for measuring uronic acid, uronic acid concentrations of wild-type (WT) K. pneumoniae B5055 were measured at different time points and at different stages of the growth curve (Fig. S3A) The levels of uronic acid associated with the bacterial pellet were strongly correlated with growth, and a standard culture optical density (OD) (OD_600_ of 0.6) was selected for analysis. The uronic acid content of pelleted K. pneumoniae B5055^Rif^ in mid-log phase (OD_600_ of 0.6) was between 68 μg/mL and 80 μg/mL. The negative controls were Escherichia coli DH5α and B5055 Δ*wza* Δ*waaF*, which contains the *wza* deletion resulting in the loss of capsule secretion, and a WaaF mutation, which leads to an LPS truncation without uronic acid (27).

A reduction of uronic acid to more than 50% of the level in B5055 was observed in some insertion mutants that demonstrated reduced string lengths and/or capsule staining (e.g., KpSC44, KpSC45, KpSC49, and KpSC5); other mutants, including KpSC42, KpSC46, and KpSC50, had uronic acid levels equivalent to that of the negative control, B5 Δ*wzb-c* Δ*waaF* (Fig. S2). The small amounts of uronic acid present in B5055 Δ*wza* were attributed to the GalA present in the LPS (29), since B5055 Δ*wza* Δ*waaF* had reduced levels of uronate compared with the capsule-deficient B5055 Δ*wzb*-*c* strain, which expresses normal LPS (31).

We concluded that the approach of using the string test and Maneval’s capsule stain identified mutants that have defects in encapsulation, and further studies were undertaken to resolve the biology of these defects.

### LPS O-antigen mutants are capsule deficient.

**FIG 2:**
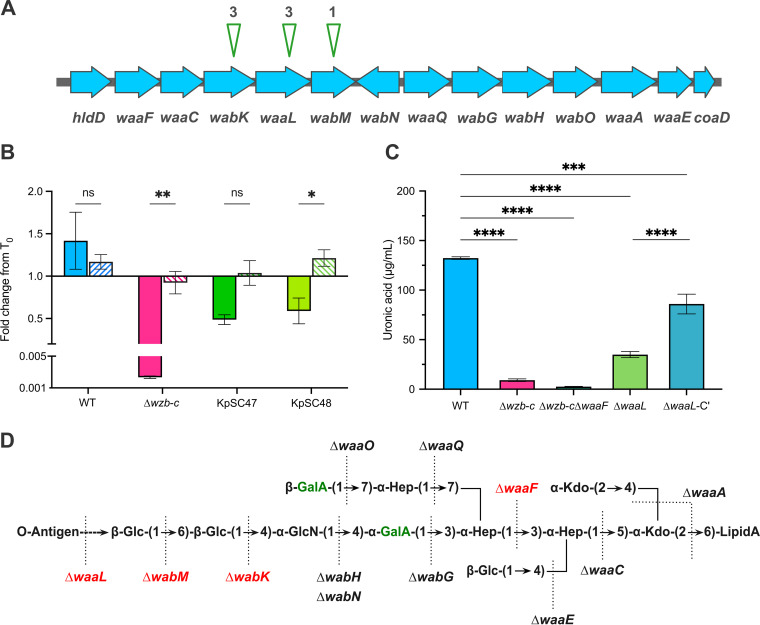
Transposon insertions into LPS-associated genes may have reduced capsules. (A) Eight capsule mutants were found to have transposon insertions within lipopolysaccharide biosynthesis genes of B5055; seven were within the *waa* operon. The insertions identified in the capsule-deficient screen are denoted by green arrowheads. (B) The sensitivity of various K. pneumoniae B5055 strains to human serum killing was evaluated in an assay where bacteria were exposed to normal human serum (NS) or heat-treated human serum (HTS) for 90 min. The viable counts were expressed as the fold change ratio from the starting inoculum for each isolate after 90 min. The values represent the mean and standard error of the mean (SEM) from 3 biological replicates and were analyzed using a two-way analysis of variance (ANOVA) with Bonferroni’s posttest adjustment for multiple testing. *, *P* < 0.05; **, *P* < 0.01; ns, not significant. (C) Uronic acid was measured in the pellets of K. pneumoniae B5505 (WT) and in selected mutants. Uronic acid was estimated in bacterial pellets of mid-log samples, and the histograms are the mean and SEM from 3 biological replicates. (D) O1 LPS structure of B5055 inferred from reference 29. The glycosyltransferases that add onto each of the sugars are denoted. The LPS insertions that affected encapsulation are shown in red. The urinate-containing sugars are shown in green. For orientation, the LPS is embedded in the membrane through lipid A (RHS), and the O antigen is added to the LHS.

**FIG 3:**
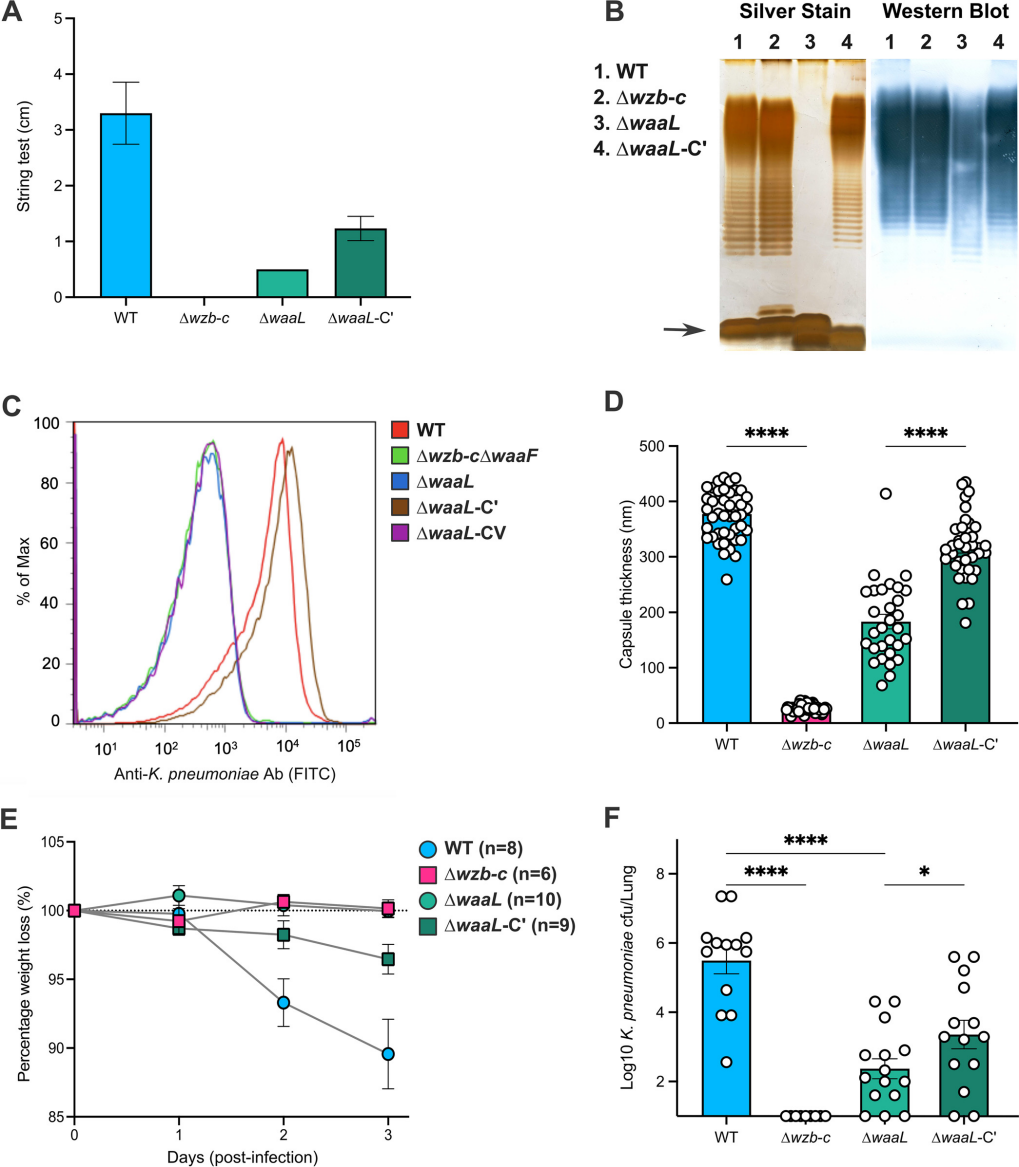
Loss of O antigen is associated with capsule loss. (A) The string test length was used to examine the relative contributions of WaaL and LPS O antigen to capsule retention. The strains tested were B5055 (WT), the capsule mutant (B5055 Δ*wzb-c*; capsule-deficient “smooth LPS”), the defined WaaL mutant (B5055 Δ*waaL*), and the complemented defined WaaL mutant (B5055 Δ*waaL-*C′) with pACYC184 expressing WaaL from the Tet^r^ promoter. The values represent the mean and SEM from 3 biological replicates. (B) Silver stain and Western blotting were used to study the LPS phenotype in each strain. LPS preparations from K. pneumoniae strains were separated by Tricine-SDS-PAGE on 15% gels and visualized by a modified silver staining method or transferred to nitrocellulose for incubation with monoclonal antibody I12, specific for the O1 LPS of K. pneumoniae (60). The Western blot of the WaaL mutant (B5055 Δ*waaL*) showed the presence of polymerized O antigen in the WaaL mutant, albeit of reduced intensity. This strain was negative by silver stain. Complementation of the WaaL mutant with pACYC184 expressing WaaL from the Tet^r^ promoter (B5055Δ*waaL-*C′) restored LPS, producing silver-stained and Western blot patterns that were similar to those in the wild-type B5055 and mutant (B5055 Δ*wzb-c*) LPS profiles. (C) Flow cytometry of difference mutants of K. pneumoniae B5055 stained with antibody I12 to determine O-antigen surface expression. The 5 strains tested were B5055 (WT), B5055 with *wzb-c* and *waaF* deleted (B5055Δ*wzb-c*Δ*waaF*), the defined WaaL deletion mutant (B5055 Δ*waaL*), and the mutant B5055 Δ*waaL-*CV complemented with the empty complementation vector (pACYC184) or with pACYC184 expressing WaaL from the Tet^r^ promoter (B5055 Δ*waaL-*C′). (D) Capsule thickness of the WaaL mutant (B5055 Δ*waaL*) and the complemented WaaL mutant (B5055 Δ*waaL-*C′) was measured by AFM. Cells were imaged in contact mode at a scan rate of 1 Hz at room temperature. The cell apex was probed during force measurements (82). The histograms represent the mean and SEM from 30 to 40 bacteria for each mutant for capsule thickness. (E and F) Groups of 10 to 12 C57BL/6 mice were inoculated intranasally with 5 × 10^4^ cells of K. pneumoniae B5055 or the various mutants and monitored for weight loss for 3 days (E). (F) After 3 days, the animals were killed and the viable count of K. pneumoniae estimated by plating of serially diluted lung homogenates onto LB agar. Groups were compared using a one-way ANOVA.

Seven transposon insertions were found within the operons required for complete LPS synthesis (Fig. 2A; Table S3), and these insertions were in the genes *wabK*, *wabM*, and *waaL*. Two independent *waaL* transposon insertion mutants, KpSC47 and KpSC48 (Table S3), were analyzed for serum sensitivity (Fig. 2B). Like the capsule-deficient mutant B5055 Δ*wzb-c*, these strains exhibited sensitivity to killing by normal human serum (i.e., containing complement), but were as resistant as B5055 to complement-inactivated normal human serum. The putatively enhanced complement sensitivity may have come from the loss of capsule: the B5055 Δ*wzb-c* mutant carries intact LPS (Fig. 3B), and this defined mutant was sensitive to killing by human complement. Uronic acid analysis of B5055 Δ*waaL* and a complemented construct (B5055 Δ*waaL*-C′) suggested that complementation increased uronic acid levels in the pelleted bacteria (Fig. 2C).

LPS consists of O-antigen polysaccharides ligated to the LPS core, which is embedded in the KpSC membrane via lipid A (Fig. 1B). B5055 has an O antigen that is serotype O1 with a type 2 *lps* core (35), with the O glycan a repeated polymer of alpha- and beta-linked galactose (Fig. S3B). The core contains 2 GalA sugars (Fig. S3C). Transposon insertions that lead to a capsule-deficient phenotype were found in 3 *lps* core synthesis genes: *wabM*, *wabK*, and/or *waaL*. These gene products act “distally” to the α-GalA sugars that have been linked with capsule retention (27, 29) (Fig. 2D, highlighted in green). In the absence of *wabM* or *wabK*, the substrate for WaaL [i.e., β-Glc (1→6)] (Fig. 2D) would not have been added. Transposon insertions in LPS biosynthesis genes that generated a reduced string suggest that there is a relationship between LPS biosynthesis and encapsulation. Mutants KpSC01, KpSC15, KpSC33, KpSC04, KpSC47, KpSC48, and KpSC52 had uronic acid levels that were <50% of that of B5055.

Capsule-deficient transposon insertions outside the *cps* and *lps* operons were observed within 12 independent capsule mutants of B5055 (Table S4). Two transposon insertions were found within the virulence plasmid pLVPK: one within the *iroC* gene and another in *pyrC* gene. In both mutants (KpSC12 and KpSC49 [*iroC* and *pyrC,* respectively]), there was no string formation, and the uronic acid concentrations were <50% of that of the wild type (Fig. S2). Mutants with insertions in *uge* (KpSC19) and *galU* (KpSC03, KpSC09, and KpSC42) had very low levels of uronic acid, equal to that of B5055 Δ*wza* Δ*waaF*, suggesting a deficiency of both LPS and capsule.

A defined WaaL (Δ*waaL*) deletion mutant was constructed in B5055; this mutant had a string length approximately 15 to 25% of that of B5055 (Fig. 3A). To ensure that the capsule phenotype in B5055 Δ*waaL* was not due to second-site compensatory mutations, the *waaL* gene was expressed from the Tet^r^ promoter present in pACYC184, and this plasmid was used to complement the defined mutant. The complementary construct was transformed into B5055 Δ*waaL* (forming B5055 Δ*waaL*-C′), and complementation doubled the string length (Fig. 3A) to approximately 50% of that of the parent B5055 strain.

The defined WaaL mutant and the complemented WaaL mutant were analyzed by silver staining, Western blotting, and by flow cytometry for LPS synthesis and surface expression/retention of the capsule. Silver-stained gels revealed the loss of the LPS “ladder” from B5055 Δ*waaL* (Fig. 3B, lane 3) and restoration of an LPS ladder in B5055 Δ*waaL*-C′ (Fig. 3B, lane 4). The O1-antigen-specific monoclonal antibody I12 (31) was used in Western blots of these preparations and revealed that the B5055 Δ*waaL* strain synthesized the high-molecular-weight O antigen (Fig. 3B, lane 3), but it was of a slightly lower molecular weight and produced at lower levels that were not seen in the silver stain gel, but which were visualized by Western blotting (Fig. 3B). The LPS core (shown by an arrow for the silver stain) appeared to be present in at least equal amounts in B5055 and the B5055 Δ*waaL* mutant. This observation suggests that the WaaL mutant synthesized the O antigen, but did not ligate the O antigen to the lipid carrier for transport to the outer membrane.

To test this hypothesis, live bacteria were stained with antibody I12 and analyzed for surface fluorescence by flow cytometry (Fig. 3C). B5055 (WT; red) was brightly fluorescent, with a mean fluorescent intensity (MFI) that was 50-fold higher than that of the negative control (B5055 Δ*wza* Δ*waaF*, a capsule mutant lacking the O antigen [green]). The B5055 Δ*waaL* mutant (blue) demonstrated I12 surface fluorescence equivalent to that of B5055 Δ*wza* Δ*waaF*. The complemented mutant (B5055 Δ*waaL*-C′ [brown]) exhibited I12 staining equivalent to that of B5055, whereas an additional control in which B5055 Δ*waaL* was complemented with the empty vector (B5055 Δ*waaL*-CV [purple]) was also negative for surface expression of the I12 epitope. The WaaL and complemented WaaL mutants of B5055 were then analyzed by AFM for capsule thickness.

To quantify how much capsular polysaccharide was retained on the surface of the B5055 Δ*wzb-c* and B5055 Δ*waaL* mutants, the capsule thickness was measured by atomic force microscopy (Fig. 3D) (Table S1). The measured capsule thickness from AFM force curves generated for the various strains revealed a capsule thickness of 377 nm on the B5055 strain, consistent with previous studies using AFM (36). The mutant B5055 Δ*wzb-c*, which lacks capsule due to the absence of the *wzb-wzc* capsule translocon, had a surface layer that radiated approximately 26 nm, the presumed length of the LPS O antigen in solution. The B5055 Δ*waaL* mutant had a mean capsule thickness of 188 nm, approximately half that of B5055, which was restored to near-wild-type levels (mean, 316 nm) by complementation (B5055 Δ*waaL*-C′). During the preparation of the bacterial samples, it was noted that the cell pellets from the wild-type B5055 and the B5055 Δ*waaL*-C′ strain were similar in size and friability and distinct from the compacted pellets of the B5055 Δ*wzb-c* and Δ*waaL* mutants (Fig. S4).

Finally, virulence tests of the parental strain B5055, the nonencapsulated B5055 Δ*wzb-c* mutant, the B5055 Δ*waaL* mutant, and the complemented (Δ*waaL*-C′) mutant were conducted. Groups of 9 to 15 C57BL/6 mice were intranasally inoculated with 5 × 10^4^ CFU, observed for 3 days, and measured for weight loss (Fig. 3E), where increased weight loss is associated with more severe disease. On day 3, the mice were killed, and lungs were removed to count viable bacteria in the tissues (Fig. 3F). The mean bacterial count in the lungs was highest in mice inoculated with B5505. The nonencapsulated mutant (B5055 Δ*wzb-c*) was not recovered from the lungs of inoculated mice. Compared with B5055, the recovery of the B5055 Δ*waaL* mutant was significantly reduced (*P* < 0.0001). In the complemented mutant, growth of the bacteria in the lungs was significantly increased (*P* < 0.016) compared with the B5055 Δ*waaL* mutant (Fig. 3E).

### Comparison of the WaaL and Wzi mutants for capsule retention.

The B5055 Δ*wzb-c* mutant lacking the functional secretion pore has no exopolysaccharide capsule, as judged by string length measurements (Fig. 3A) and reduced uronic acid analysis (Fig. 2C); the small amounts of urinate in the Δ*wzb-c* mutant are likely contributed by the galacturonic acid present in the LPS core (Fig. 2D). The B5055 Δ*waaL* mutant retained less than 50% of the capsule polysaccharide on its surface (Fig. 3D), suggesting that the distal chains of the O antigen provide an anchor or stabilizer for the capsule. To address previous observations that Wzi contributes to capsule retention (30, 37), we constructed two further mutants, B5055 Δ*wzi* and B5055 Δ*waaL* Δ*wzi*, to determine whether Wzi was acting in concert with WaaL to retain capsule.

**FIG 4:**
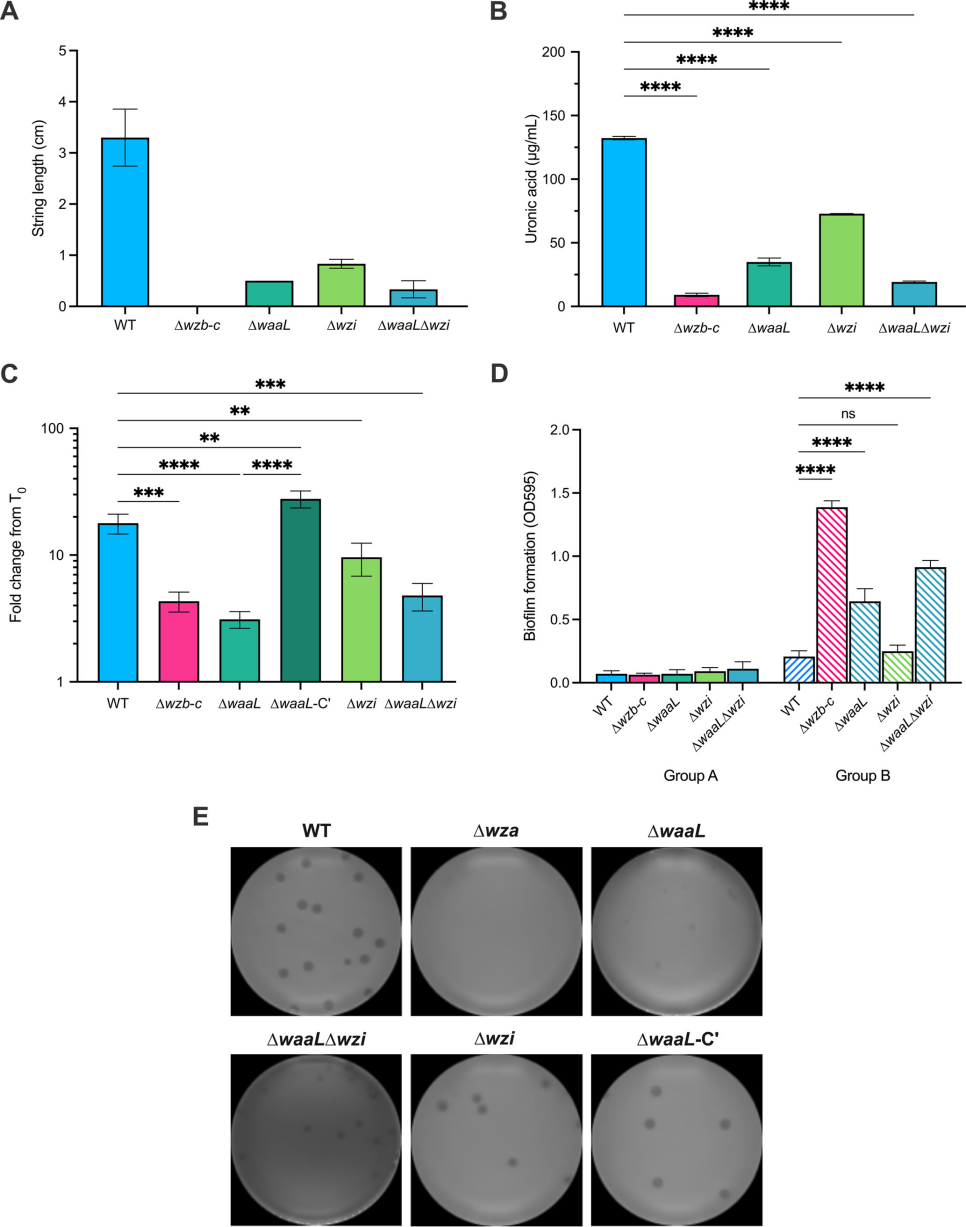
Contribution of WaaL and Wzi to capsule retention in K. pneumoniae. To examine the relative contributions of Wzi and LPS O antigen to capsule retention, the wild-type B5055 strain, single mutant strains (Δ*wzb-c*, Δ*wzi*, Δ*waaL*, and complemented Δ*waaL*) (C and E), and double mutant strain (Δ*waaL* Δ*wzi*) were analyzed for (A) string test length, (B) uronic acid levels associated with the bacterial pellets, and (C) survival in unheated human serum for 90 min, as previously described. The mutants were also tested for (D) formation of static biofilms. In panels A to C, the histograms represent the mean and SEM from 3 biological replicates, and the different strains were compared using a one-way ANOVA. For biofilm measurements (D), B5055 and the deletion mutants were tested with (group B) and without (group A) transformation with a plasmid expressing MrkH (59). MrkH is a cyclic-di-GMP-sensitive transcriptional activator of Mrk fimbria expression that has been naturally deleted from B5055, resulting in poor biofilm formation. The bacteria were added to the wells under optimized conditions and left for 24 h, and the wells were stained with crystal violet. The binding of crystal violet was determined using acetic acid solubilization, and absorbance was measured at 595 nm in a spectrophotometer. The mean and SEM from each sample (3 biological replicates, 5 technical replicates of each) were compared with those of the parent WT strain by one-way ANOVA (E). The strains were also tested for their sensitivity to a K2-specific bacteriophage, RAD2, in a plaque assay (39). The number and type of plaques reflect the sensitivity of each of the mutants to RAD2.

Again, the capsule translocon mutant (Δ*wzb-c*) generated no string, while the WaaL and Wzi single mutants and WaaL Wzi double mutant all showed reduced string lengths compared with B5055 (Fig. 4A). The level of uronic acid associated with the cell pellet in the Wzi mutant was greater than that for the WaaL mutant, and the WaaL Wzi double mutant had further reduced levels of urinate compared to the WaaL mutant (Fig. 4B). In the human whole-blood assay (Fig. 4C), growth of B5055 was observed over the incubation period in blood. In comparison, there were declines in the bacterial counts of the capsule translocon mutant (Δ*wzb-c*) and the O-antigen ligase mutant (Δ*waaL*) in whole human blood, while there was growth in the complemented WaaL mutant (Δ*waaL-*C′). The loss of Wzi (Δ*wzi*) had limited impact on the survival of the bacteria, but net growth was not observed, and the double mutant without WaaL and Wzi had a loss-of-viability phenotype similar to that of the WaaL mutant.

### Biofilm formation and bacteriophage sensitivity.

The impact of mutation of WaaL and loss of O antigen from LPS on biofilm formation was then investigated (Fig. 4D). K. pneumoniae B5055 is a poor biofilm former because it lacks an intact copy of the Mrk activator MrkH (38). B5055 and the various mutants were complemented with a pACYC184 plasmid carrying MrkHI, expressed from the endogenous promoter (p*mrkHI*) (38). The isolates were then analyzed for static biofilm formation at 24 h postinoculation, using crystal violet staining (38) (Fig. 4D). The data were divided into two groups: those without p*mrkHI* (group A) and those with the plasmid expressing the positive regulator of the Mrk fimbriae (group B). Biofilm formation in B5055 was strongly increased by transformation with the plasmid expressing MrkHI. The highest level of biofilm formation was in the positive control, which lacks the capsule translocon. The WaaL mutant also showed statistically significant increases in biofilm formation compared with the wild type (WT), whereas the increase in biofilm formation seen with the Wzi mutant compared with the wild type was smaller and not statistically significant.

Finally, the sensitivity of the WaaL and Wzi mutants to bacteriophage RAD2 was examined. RAD2 requires the K2 capsule as a receptor for infection (Fig. 4E). Previous studies have shown that the B5055 Δ*wza* Δ*waaF* mutant lacking the O antigen and the *wzb-c* mutant lacking the secretion pore are completely phage resistant (39). As expected, clear RAD2 plaques formed on B5055, but not on the Wza capsule-deficient mutant. The WaaL mutant show a reduced number of turbid plaques, suggesting inefficient binding, whereas infection of the complemented WaaL mutant produced normal plaques. Normal plaques were formed by RAD2 on the Wzi mutant, but the Wzi WaaL double mutant had a phenotype similar to that of the WaaL mutant. These data suggest that WaaL is required for full sensitivity to RAD2, a phage specific for the K2 capsule.

## DISCUSSION

The production and retention of an exopolysaccharide capsule are critical steps in the virulence of many bacterial pathogens, and while there is a good understanding of how the capsular polymers are synthesized and secreted (40), much less is understood about the retention of the capsule on the bacterial cell surface. In Gram-negative bacteria, some of the capsule polymers may be linked to the bacterial outer envelope through a lipid carrier. In so-called “group 2” capsules (41), a poly-2-keto-3-deoxyoctulosonic acid (poly-KDO) linker is attached to a lysophosphatidylglycerol lipid moiety thatcan embed within the cell envelope (28, 42). However, this may not be true for all the individual capsular carbohydrate polymer chains or for group 1 capsules. Better resolution of the mechanism of capsule retention might assist the development of strategies to deencapsulate pathogens, rendering them much more sensitive to innate defense mechanisms, and hence less virulent.

Our studies sought to identify genes involved in encapsulation that sat within and outside the major capsule locus, using transposon mutagenesis of a heavily encapsulated, virulent strain of the opportunistic pathogen K. pneumoniae, known as B5055. B5055 is mouse virulent (43), expresses O1 LPS and K2 capsule, and was first described by Goslings and Snijders (44). Mutations that might affect encapsulation could act at various levels—there are biochemical processes involved in capsule polymer synthesis, capsule polymer secretion, and capsule retention that might all affect the capsule phenotype of a bacterial pathogen. In a transposon screen using simple, high-throughput phenotypic approaches (e.g., measuring string length supported by Maneval staining for capsule), it was not possible to differentiate the cause of deencapsulation without further analysis. This was enabled by uronic acid assays, staining reactions, flow cytometry, and AFM. The biological effects of these mutations were analyzed in bacterial survival assays *in vitro* and *in vivo*.

A total of 8,400 colonies were screened for capsular defects following random transposon mutagenesis of B5055, from which 53 mutants were further analyzed for string length and uronic acid production. One mutant produced much longer strings. While the string test is not fully understood, nonencapsulated mutants of B5055 form very short strings compared with the parent, B5055. Thirty-nine mutants that had reduced string test values and reduced urate production were analyzed by Y-linker PCR for the insertion site of the transposon. The query sequences showed more than 98% nucleotide identity to the homologous genes present in the “reference” K. pneumoniae MGH 78578 genome or the *cps* gene cluster of the K. pneumoniae Chedid strain (also an O1:K2 strain), or the *waa* operon of K. pneumoniae 52145 (O1:K2). Some genes associated with reduced string lengths were independently mutagenized multiple times by albeit nonsibling transposon insertions. After the screen and Y-linker analysis, insertions were identified in 22 different open reading frames that yielded a capsule-defective phenotype in K. pneumoniae B5055, including 6 genes (with a total of 19 independent insertions) known to be directly involved in capsule synthesis, such as *wza* and *wzc*, which form part of the translocon (see Table S2 in the supplemental material), providing a validation of the approach. Four insertions were found in *lps* biosynthesis gene cluster, 10 genes were found outside both *cp*s and *lps* operons, but in the bacterial chromosome, and 2 genes were found in the plasmid pLVPK.

One mutant, KpSC31, displayed an increased string length. This mutant carried a transposon insertion in *lpxM*/*msbB*, which codes for lipid A lauroyl acyltransferase according to the Raetz pathway (45). During the final steps of lipid A biosynthesis in E. coli, acyloxyacyl moieties are generated by addition of lauroyl and myristoyl residues to the distal glucosamine unit (45). Mutation in the *lpxM* gene in E. coli leads to attenuation in the mutants for activation of human macrophages (46), although the gene is not essential for growth (47), *lpxM* expression is a virulence trait in the murine model of E. coli pathogenicity (48). In Salmonella, mutations in the *lpxM* gene render the mutants less lethal than the wild-type strain in animal septic shock models (49, 50). In K. pneumoniae B5055, mutation in *lpxM* renders the bacterium attenuated for growth and less lethal in mouse pneumonia models (51). The reasons why the *lpxM* insertion mutant gave rise to a grossly increased string length were not explored, but there was no increase in uronic acid associated with the mutant, suggesting that increased string length may not always be reflective of increased encapsulation, but might result from, e.g., the strength of interactions between carbohydrate polymers and the outer envelope through lipid A, which is hexa-acylated by LpxM, or the length of the capsule polymer chains.

This study suggests that generation of wild-type levels of bacterial encapsulation in the archetypical encapsulated Gram-negative bacterium K. pneumoniae is a complex phenomenon involving genes directly required for capsule biosynthesis and numerous other genes outside the capsule locus, such as *degP*, coding for a periplasmic protease (52), *tepK*, coding for a putative efflux/permease protein with homology to the 14-transmembrane-domain DHA2 family of the major facilitator superfamily (MFS) (53), and *ompW*, an 8-stranded beta-barrel protein that provides E. coli with enhanced resistance to phagocytosis and complement resistance (54).

The capsule-deficient mutants that contained transposon insertions in the *lps* operons were in enzymes that synthesized the relatively conserved LPS carbohydrate core and typically core synthesis distal to the GalA (galacturonic acid) residue(s)—in *wabK* and in *wabM* (Fig. 2D). Previous studies suggested that the capsule-retentive interaction between LPS and capsular polysaccharides was mediated through ionic interactions between capsule “fibrils” and the LPS core galacturonic acids (GalA), a distal sugar on the branch of the LPS core carbohydrate that is also present in the “unbranched” LPS core (29). Our data suggested that the interaction between the attached polymerized O antigen and the capsule monomers stabilizes capsule retention.

Three independent capsule-deficient mutants were identified with insertions within *waaL*, the O-antigen ligase that attaches O antigen to the completed LPS core (55); according to the published structures of the K. pneumoniae O1 LPS, each of these mutants retained at least 1 GalA in the rough LPS that was synthesized. WaaL mutants are attenuated for *in vitro* inflammation (56) and *in vivo* in a murine septicemia model (29). To explore the relationship between O antigen and encapsulation further and to remove the potential confounding effects of transcriptional polarity from the transposon, a defined “gene-gorged” mutant with *waaL* mutation was constructed and complemented. This defined *waaL* mutant phenocopied the transposon insertion into *waaL* and was capsule deficient. Interestingly, the defined *waaL* mutant continued to synthesize the LPS O-antigen polymer, as determined by Western immunoblotting. Flow cytometry showed that this O antigen was not on the bacterial surface, however, and that the significant loss of encapsulation in the *waaL* mutant, defined by AFM, was restored when the *waaL* mutant was complemented (Fig. 3D; Table S1). The *waaL* mutant was attenuated for growth in mice compared with the B5055 parent strain, but retained virulence for mice in a pneumonia model beyond that of the B5055 Δ*wza* nonencapsulated strain. The relative roles of Wzi, the surface lectin involved in capsule assembly (30, 57), were also assessed by mutagenesis. The loss-of-capsule phenotype was greater in the WaaL mutant than in the Wzi mutant, and the WaaL mutant, like the capsule-deficient mutant (Δ*wzb-c*), did not support efficient growth of the K2-specific RAD2 bacteriophage.

The apparent paradox, whereby increased biofilm formation was reported in nonencapsulated mutants of K. pneumoniae (58), is consistent with our observations that the WaaL mutant formed larger biofilms. In K. pneumoniae, biofilm formation on plastic substrates is dependent on Mrk fimbriae (59), and B5055, the strain used in this study, does not naturally form biofilms because it lacks the transcriptional activator of the Mrk fimbriae, MrkH (59). This gene was provided as a plasmid to test whether the WaaL mutation affected biofilm formation. Previous studies had suggested that Mrk-mediated biofilm formation was enhanced when capsule was deleted (20, 60). The mutation of WaaL had a greater positive impact on biofilm formation than the loss of Wzi, and addition of a Wzi mutation to the WaaL mutation further increased biofilm formation, suggesting that the WaaL and Wzi functions may not be redundant with respect to biofilm production. The reason for the increase in biofilm when encapsulation is reduced is likely explained by greater Mrk fimbrial access to the polycarbonate substrate on which the biofilms formed, though the mechanoproperties of the capsule are also altered by fimbriae (61, 62). These data suggest that the degree of encapsulation, and hence, inversely, biofilm formation, may be regulated by one or more of the multiple mechanisms known to increase the chain length of the O antigen (63).

**FIG 5:**
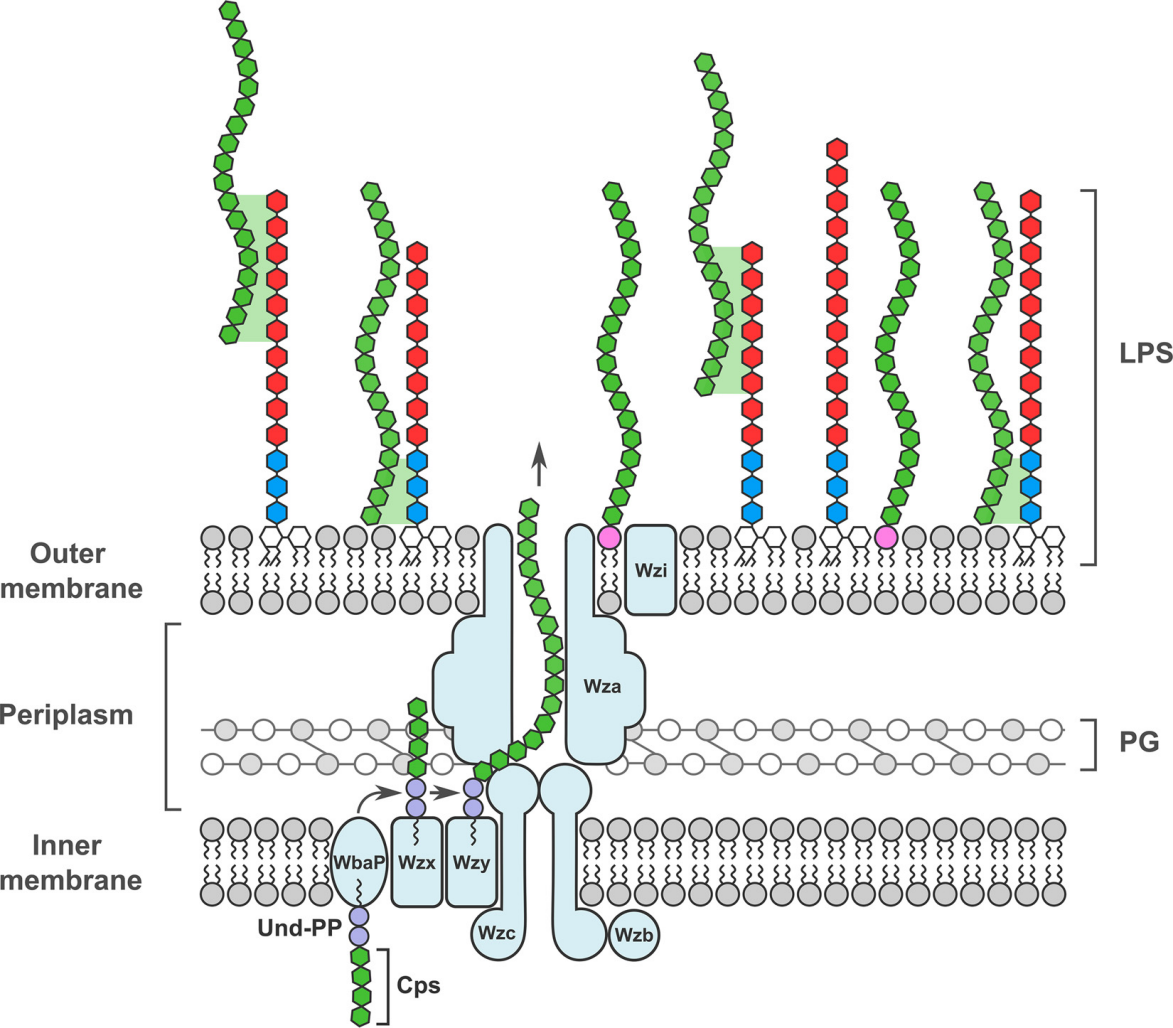
Amended model for capsule retention by K. pneumoniae. The association between the O antigen in the LPS and the polysaccharide capsule occurs through unresolved forces and possibly simple entanglement of the fibrils. The O antigen is shown in red and the core oligosaccharide in blue. Some capsule types may use an acylated carrier (pink) to link the capsule fibril to the bacterial surface. The ligation of LPS O antigen is required for maximal capsule retention, and Wzi plays a minor role in capsule retention, possibly through its properties as a lectin (57).

These data very strongly suggest that capsule retention in *Klebsiellae* is stabilized by an interaction between surface oligosaccharides present in the O-antigen component of the LPS and the individual capsule fibrils (Fig. 5). An alternate, albeit less likely, hypothesis that WaaL directly ligates the capsule polymer to the core of LPS, matured by WabK and WabM, was not investigated. To rule out effects of accumulated O antigen on *cps* transcription, we quantified the transcription of key *cps* genes in the wild type and WaaL mutant strains of B5055. There were no obvious differences in *cps* transcription in the wild-type B5055 and WaaL mutant B5055 (Fig. S5).

The interaction between the LPS O carbohydrate and the capsule monomer could occur physically, through entwining of capsule polymer fibrils with fully polymerized and ligated O antigen, or the LPS may help stabilize long polymers, anchored into the outer membrane through a covalently associated glycolipid moiety, or noncovalently via the core GalA sugars. Finally, while O-antigen–capsule monomer interactions would not explain capsule retention in some Gram-negative pathogens, like Neisseria meningitidis and Haemophilus influenzae, which express an oligosaccharide LPS only (64, 65), our findings suggest that LPS-based vaccines that induce antibodies that drive complement-mediated lysis or phagocytosis of K. pneumoniae through binding to O antigen may have an additional protective benefit through reducing encapsulation. Moreover, the data suggest that *Klebsiella* biofilm formation, which is seen as a key virulence trait that is mediated through Mrk fimbriae and important in colonization of plastics (59), may be regulated through processes that control the length of the O antigen.

## MATERIALS AND METHODS

### Strains.

Klebsiella pneumoniae B5055 is a mouse-virulent, O1:K2 strain of K. pneumoniae and was the generous gift of D. Hansen from the Statens Serum Institute, Copenhagen. The derivates used in this study are shown in Table 1. The Escherichia coli S17-1 λ*pir* strain containing the pUT vector harboring the transposon miniTn*5Km2* was used in the transposon mutagenesis (32). The capsular mutant B5055 Δ*wzb-c* was the result of site-directed mutagenesis of the *wzb* and *wzc* genes of CPS synthesis using the Lambda Red recombinase system, as described in reference 31.

**TABLE 1:** Bacterial strains and plasmids used in this study

Strain or plasmid	Characteristic(s)^*a*^	Reference or source
Strains		
*K. pneumoniae* B5055	Wild type, encapsulated K. pneumoniae O1:K2	33; data not shown
*K. pneumoniae* B5055^Rif^	Rifampin-resistant derivative of B5055	This study
*K. pneumoniae* B5505Δ*wzb-c*	Defined *wzb*-*wzc* functional capsule translocon deletion of B5055, capsule deficient, wild-type O1 LPS	20
*E. coli* DH5*α*	Nonencapsulated, deep rough mutant of E. coli K-12, recombination-impaired cloning strain	Thermo Fisher Scientific
*E. coli* S17-1 λ*pir*	Donor strain for conjugational transfer of transposons, carries suicide plasmid pUT harboring mini-Tn*5Km2* transposon, Km^r^	58
*K. pneumoniae* B5505 Δ*wza* Δ*waaF*	B5505 Δ*wza* carrying a mutation in WaaF, no capsule or LPS O antigen	31
*K. pneumoniae* KpSC01-KpSC53	Tn*5* transposon insertions into B5055^Rif^	This study
*K. pneumoniae* KpSC47, KpSC48	Independent Tn*5* insertions into *waaL* gene of B5055	This study
*K. pneumoniae* B5055 Δ*waaL*	B5055 with *waaL* deleted	This study
*K. pneumoniae* B5055 Δ*waaL-*C′	B5055 Δ*waaL* carrying pACYC184::*waaL*	This study
*K. pneumoniae* B5055 Δ*waaL-*CV	B5055 Δ*waaL* carrying pACYC184	This study
*K. pneumoniae* B5055 Δ*wzi*	B5055 with *wzi* deleted	This study
*K. pneumoniae* B5055 Δ*waaL* Δ*wzi*	B5055 with *waaL* and *wzi* deleted	This study
*K. pneumoniae* KpSC01–KpSC53	Independent Tn*5* insertions of B5055^Rif^ having a string test difference	This study
Plasmids		
pACYC184	Medium-copy-no. vector, Cm^r^ Tc^r^	75
pACYC184::*waaL*	pACYC184 containing B5055 *waaL* gene inserted under control of TetR promoter, Cm^r^	This study
pGEM-T Easy	High-copy-no. vector, Ap^r^	Promega
pACBSR	Mutagenesis plasmid, carries genes for I-SceI endonuclease and Lambda Red recombinase	73
pKD4	Vector containing Km^r^ gene, Ap^r^ Km^r^	74

a Ap^r^, ampicillin resistance; Cm^r^, chloramphenicol resistance; Km^r^, kanamycin resistance; Tc^r^, tetracycline resistance.

### Transposon mutagenesis.

The mini-Tn*5* transposon was delivered from a mobilizable pUT plasmid, containing the π protein-dependent origin of plasmid R6K and harboring the Tn*5* transposase, which sits outside the transposable resistance cassette. Since K. pneumoniae B5 carries no innate antibiotic resistance that could be utilized for selection, a rifampin-resistant derivative of B5055 was used. B5055^Rif^ was created by plating the parent strain (wild-type B5055) on a gradient of rifampin and selected from spontaneously resistant mutants (66). Transposon mutants were created by conjugation of E. coli S17-1 λpir strains containing pUT vector harboring the transposon miniTn*5Km2* with Klebsiella pneumoniae strain B5055^Rif^, serotype K2.

### String test.

Each mutant was cultured for single colonies for 18 h on LB agar. The formation of a string was determined using a bacteriological loop. The maximal length of the string formed was measured in centimeters, recorded from at least three independent colonies.

### Maneval’s stain.

A drop of 1% (wt/vol) Congo red dye was placed on a glass slide, and a loop of bacteria was mixed gently into the drop of dye. The stain was air dried and counterstained with Maneval’s stain (acetic acid and acid fuchsin) for 2 to 3 min. The slide was washed in distilled water, air dried, and examined under oil immersion. The degree of encapsulation was compared with those of B5055 and B5055 Δ*wzb-c* (67).

### Uronic acid quantification.

The measurement of uronic acid for quantification of CPS in K. pneumoniae was performed as described previously (68). The modified carbazole assay uronic acid measures uronic acids, including glucuronic acid, one of the four sugars present in the CPS of K. pneumoniae K2 (69, 70). Uronic acid is present in the LPS of K. pneumoniae B5055; as an O1:K2 isolate, the percentage of glucuronic acid in K. pneumoniae K2 strain B5055 should be 21.6% by weight of CPS (29, 34). The relationship between glucuronic acid and absorbance at 520 nm was linear up to a concentration of 200 μg/mL. The measurements were performed in microtiter plates (71). Single colonies were inoculated into LB broth and incubated overnight at 37°C with shaking. Overnight cultures were diluted in 1/50 LB broth and grown at 37°C with shaking to obtain mid-log cultures (OD_600_ = 0.6). Three biological replicates were taken for each mutant, culture density was measured at 600 nm, and CPS extraction was carried out by the method of Campos et al. (68). The amount of uronic acid was determined by measuring absorbance at 520 nm following addition of 0.15% 3-hydroxy-diphenol in 0.5% NaOH (68–70), and the readings were compared with a standard curve of uronic acid.

### Transposon mutagenesis.

The mini-Tn*5* transposon was delivered from a mobilizable pUT plasmid containing the π protein-dependent origin of plasmid R6K. Since K. pneumoniae B5 carries no innate antibiotic resistance that could be utilized for selection, a rifampin-resistant derivative of B5055 was used. B5055^Rif^ was created by plating the parent strain (wild-type B5055) on a gradient of rifampin and selected from spontaneously resistant mutants (66). Transposon mutants were generated by conjugation of E. coli S17-1 λ*pir* strains containing the pUT vector harboring the transposon miniTn*5Km2* with Klebsiella pneumoniae strain B5055^Rif^, serotype K2. Mid-log cultures of donor (E. coli S17-1 λ*pir* harboring suicide vector pUT-mini-Tn*5Km2*) and recipient (K. pneumoniae B5055^Rif^) were mixed in a 1:1 ratio in a final volume of 1 mL. The conjugation mixture was then centrifuged, resuspended in 0.1 mL LB, and grown on LB agar for 6 h. Bacterial lawn growth was resuspended in LB, diluted 1:100, and replated onto LB agar containing kanamycin and rifampin. A total of 8,400 kanamycin- and rifampin-resistant colonies were streak diluted on LB agar containing kanamycin and subsequently stored in LB containing 10% glycerol and kanamycin at −70°C until required. The sequences flanking the transposon insertions were amplified by Y-linker ligation PCR (72).

### Y-linker mapping of transposon insertions.

The Y-linker method uses a Y-shaped linker that was designed to have a 3′ overhang complementary to the “sticky end” generated by the restriction enzyme digestion of the chromosome (72). Electrophoretic analysis of the PCR amplicons following Y-linker ligation PCR determined that for the mutants selected, the transposon had inserted only once into the genome. The PCR product was sequenced to identify the insertion site.

### Construction of clean deletions.

Gene gorging (73) was used to construct clean deletion mutants in K. pneumoniae B5055 using a two-plasmid system. The mutagenesis plasmid pACBSR carries the genes for I-SceI endonuclease and the λ Red recombinase, under the inducible control of the arabinose promoter (74). Approximately 0.5-kb regions flanking the upstream and downstream sequence of the target gene were PCR amplified using the I-SceI primers on the K. pneumoniae B5055 strain as the template. The kanamycin resistance gene was amplified from pKD4, using KanF and KanR primers, such that the resulting product contains fragment length polymorphism recombinase target (FRT) sites to permit subsequent excision of kanamycin cassette. The excision of the kanamycin cassette from the chromosome of the deletion mutants used the helper plasmid pCP20. The temperature-sensitive plasmid pCP20 was removed by incubation at 42°C and the genotypes confirmed by PCR.

### Complementation of WaaL.

The gene encoding WaaL was amplified from K. pneumoniae B5055, ligated into pGEM-T Easy, and sequenced. The constructs were gel isolated after restriction enzyme digestion and inserted within either the tetracycline or chloramphenicol resistance-encoding genes of pACYC184 (75) via unique restriction sites. In pACYC184::*waaL*, the plasmid contained B5055 DNA that was transcribed from the Tet^r^ promoter (i.e., in the same direction as the antibiotic gene). The constructs were maintained in cells using chloramphenicol.

### Human serum killing assay.

Human serum (10% sera in phosphate-buffered saline [PBS]) was aliquoted into 10-mL tubes, and duplicate samples were tested in each assay. One aliquot (5 mL) was heat treated by incubation at 56°C for 30 min (HTS), while the other aliquot (5 mL) was kept on ice (NS), whereafter 500 μL of appropriate serum was aliquoted into a 24-well plate, labeled, and kept on ice until bacteria were added. One milliliter of bacterial culture at an OD_600_ of 0.6 was centrifuged at 13,000 × *g* for 5 min. The pellet was resuspended in 1 mL PBS and diluted to obtain 1 × 10^6^ CFU/50 μL (time zero [*T*_0_] sample) and kept on ice. A 50-μL aliquot of the starter culture (*T*_0_) was added to the serum and incubated at 37°C for 90 min with shaking (180 rpm). The various dilutions of the starter culture, HTS samples, and NS samples were spread plated on LB agar plates with antibiotics and incubated at 37°C overnight.

### Whole-blood assay.

K. pneumoniae B5055 and mutants were grown to mid-log phase. The bacterial cells were pelleted by centrifugation at 13,000 × *g* for 5 min. The cells were washed in PBS and diluted to make a final CFU of ~5 × 10^7^/mL. Sterile Vacuette tubes containing lithium heparin (Greiner Bio-One, Frickenhausen, Germany) were used to collect murine or human blood, which was used on the day of collection. To study phagocytosis, 100 μL of diluted bacterial culture was added to 300 μL of whole heparinized blood, and the mixture was incubated at 37°C for 3 h with rotation. Samples were plated for viable bacterial counts before and after incubation.

### SDS-PAGE and silver stain.

LPS samples were prepared using the proteinase K-hot phenol method described by Marolda et al. (76). Samples were separated with Tricine-SDS-PAGE using 15% polyacrylamide gels, followed by LPS staining and visualization with 0.1% (wt/vol) silver nitrate, as described by Kittelberger and Hilbink (77).

### Method for Western blotting.

The LPS O1 antigen was detected by immunoblotting using the monoclonal antibody I12, as described by Clements et al. (60). Following SDS-PAGE, samples were transferred to nitrocellulose membrane. The primary antibody was used at a dilution of 1:1000. A goat anti-mouse horseradish peroxidase (HRP) conjugate (Bio-Rad) was used as the secondary antibody at a dilution of 1:10,000. Detection was by colorimetric development with TMB (3,3′,5,5′-tetramethylbenzidine) membrane peroxidase substrate (Seracare).

### Flow cytometry.

Monoclonal antibodies F1 and I12 (raised against outer membrane proteins [OMPs] of K. pneumoniae B5055 Δ*wza*) were used for detection of capsule and LPS by flow cytometry. The bacterial strains were grown overnight in LB broth at 37°C at 180 rpm. One-milliliter aliquots of the overnight culture were centrifuged at 13,000 × *g* for 7 min to obtain the cell pellet, which was then washed and resuspended in 1 mL PBS. One hundred-microliter aliquots were centrifuged to pellet the bacteria, followed by removal of the PBS. The pellet was resuspended in (1:50) F1 or (1:250) I12 antibody in 0.2% (wt/vol) bovine serum albumin (BSA) in PBS and incubated at 37°C for 60 min. The samples were centrifuged to remove the supernatant, followed by washing three times with PBS. The pellet was resuspended in 100 μL of 1:300 fluorescein isothiocyanate (FITC)-labeled anti-mouse Ig (λ+K) in 0.2% BSA in PBS or anti-mouse IgG (only for F1 antibodies) in 0.2% BSA in PBS. The samples were incubated at 37°C in the dark for 60 min. The supernatant was removed after centrifugation of the samples, followed by washing three times with PBS. The cells were gently resuspended in 200 μL of 4% (vol/vol) formaldehyde and incubated at room temperature for 20 min to kill the bacteria. The sample was centrifuged to remove the formaldehyde supernatant, followed by washing in PBS. The cells were resuspended in 100 to 500 μL PBS and analyzed by fluorescence-activated cell sorting (FACS) using FACSort (BD Biosciences, USA) with the appropriate voltage and compensation settings, which were determined by using reference samples (bacteria incubated without primary antibodies).

### Atomic force microscopy.

Strains were maintained on Luria-Bertani (LB) agar at 37°C. LB broth samples inoculated with these cultures were grown for 16 h at 37°C while shaking (180 rpm). Stationary-phase cells were then harvested by centrifugation (10 min at 3,500 × *g*) and washed twice with Milli-Q water (18.2 MΩ cm^−1^). The final concentration of bacterial cells in Milli-Q water was approximately 2 × 10^8^ CFU mL^−1^. Cells were adhered to gelatin-coated glass slides to ensure immobilization for AFM measurements Substrate rigidity is a requirement when measuring cell indentation to ensure that only cell compression contributes to the measurement. The gelatin coating method is described elsewhere (78). Wang et al. (78) found that there is no measurable effect of gelatin deformability on the force profiles of the bacteria. All mechanical measurements were performed within 8 h of removal of the bacteria from growth medium. Bacterium-coated slides were immersed in 10 mM HEPES buffer (pH 7.4) and kept at rest within the calibrated atomic force microscope for at least 40 min before measurements commenced, as described previously (78). AFM measurements were performed using an MFP-3D instrument (Asylum Research, Santa Barbara, CA). Silicon nitride cantilevers were purchased from Bruker (MLCT, Camarillo, CA), with a nominal spring constant of 0.02 N m^−1^ and probe radius of 20 nm (according to the manufacturer’s specifications). Cantilever spring constants were determined using the thermal tune method (79) included in the MFP-3D software. Calibrated spring constants were within the range of 0.016 to 0.024 N m^−1^. All cantilevers used were from the same batch. All tips were cleaned in a BioForce UV/ozone cleaner (BioForce Nanosciences, Inc., Ames, IA) before use. Photodetector sensitivity was measured on a clean silica slide prior to force measurements (80). The slope of the constant compliance region of the force curves obtained was used to convert the deflection, *d*, in millivolts to nanometers. The cantilever defection was then converted into a force, *F*, according to Hooke’s law, *F = k × d*, where *k* is the force constant of the cantilever (81). Cells were imaged in contact mode at a scan rate of 1 Hz at room temperature (typically 20°C). Trace and retrace were monitored to locate the true apex of cells, and force curves were measured at different locations along it. The cell apex was probed during force measurements rather than the cell periphery, which has a high degree of curvature that makes quantification of mechanical properties difficult (82). Imaging was repeated after each collection of force curves to ensure no change in cell morphology had occurred. Force curves were acquired at a loading rate of 600 nm s^−1^. For each cell type, force curves were collected on at least 30 cells from 3 different preparations consisting of at least 7 cells. Eleven force curves were measured along the apex of each cell, and the median curve was selected for analysis and was only included in the analysis if the force curves showed good reproducibility from location to location.

### Bacterial cell indentation and force curve analysis.

As demonstrated previously (36, 83), the force curves that result from the indentation of K. pneumoniae cells are comprised of two distinct regions, linear and nonlinear, when working at low electrolyte concentrations. The indentation depth at which the linear portion of the force curve begins provides an estimate of the bacterial capsule thickness, where the contact point (i.e., zero indentation) is defined as the onset of cantilever deflection since long-range noncontact double-layer interactions are negligible in this study. (The electrolyte concentration is 10 mM, which corresponds to a Debye length of ~3 nm.)
