# Supplementary material for: LPS O Antigen Plays a Key Role in Klebsiella pneumoniae Capsule Retention
Source: Microbiol Spectr. 2022 Aug 1;10(4):e01517-21. doi: 10.1128/spectrum.01517-21 (PMC9431683; doi:10.1128/spectrum.01517-21)
Supplement: Supplemental file 1 — Supplemental material. Download spectrum.01517-21-s0001.pdf, PDF file, 0.9 MB [file spectrum.01517-21-s0001.pdf]

Supplementary Figure 1A

Workflows for identifying mutants with reduced capsules

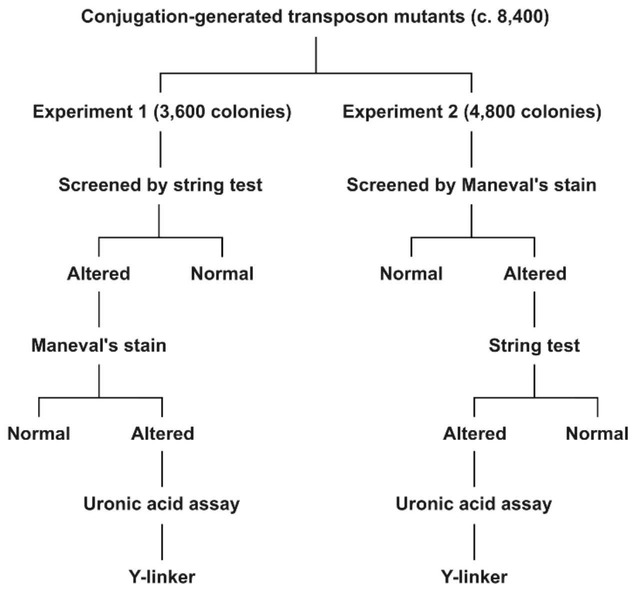

Supplementary Figure 1B

String test results for B5055 Tn*5* mutants

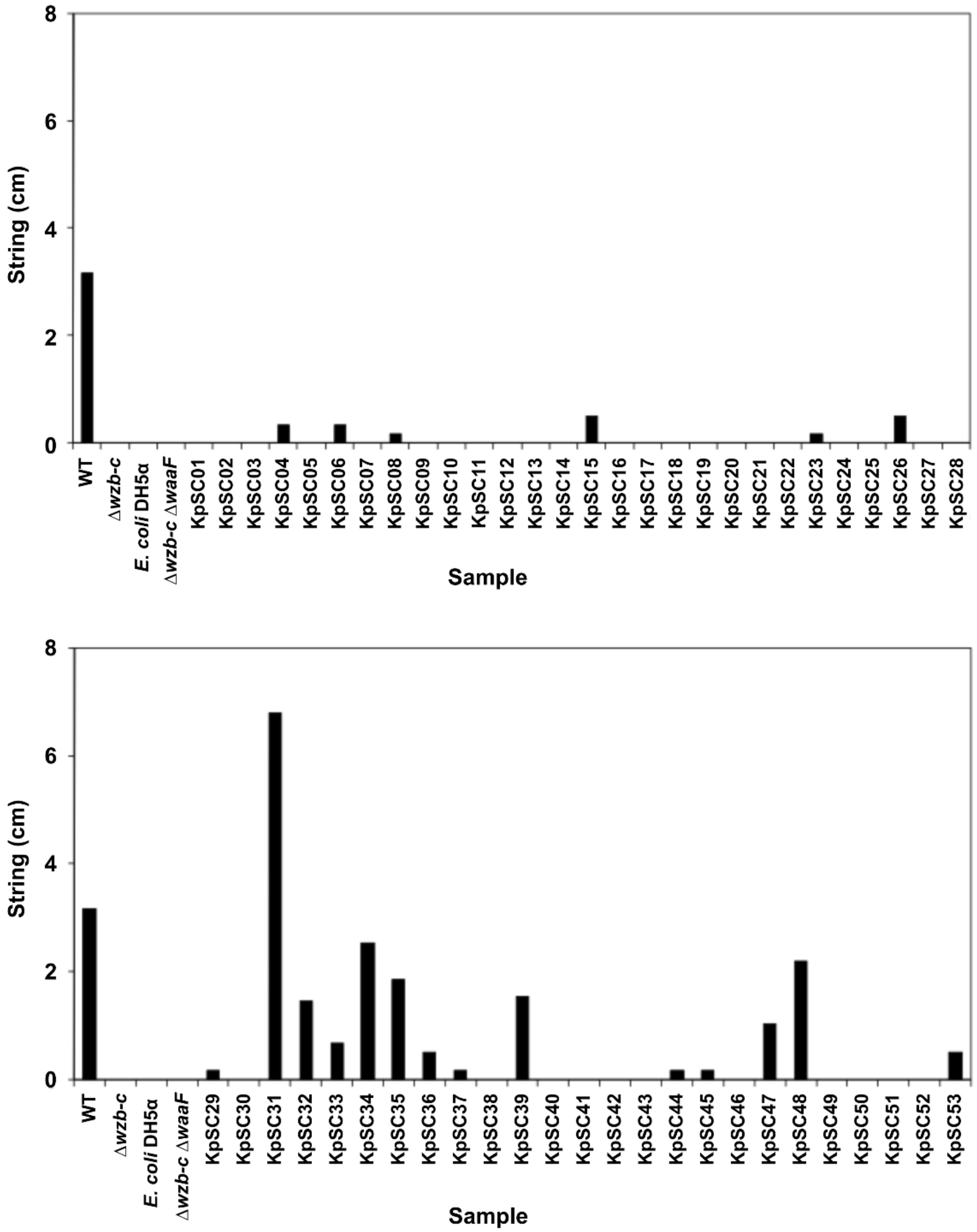

Supplementary Figure 2

Uronic acid production by string test mutants

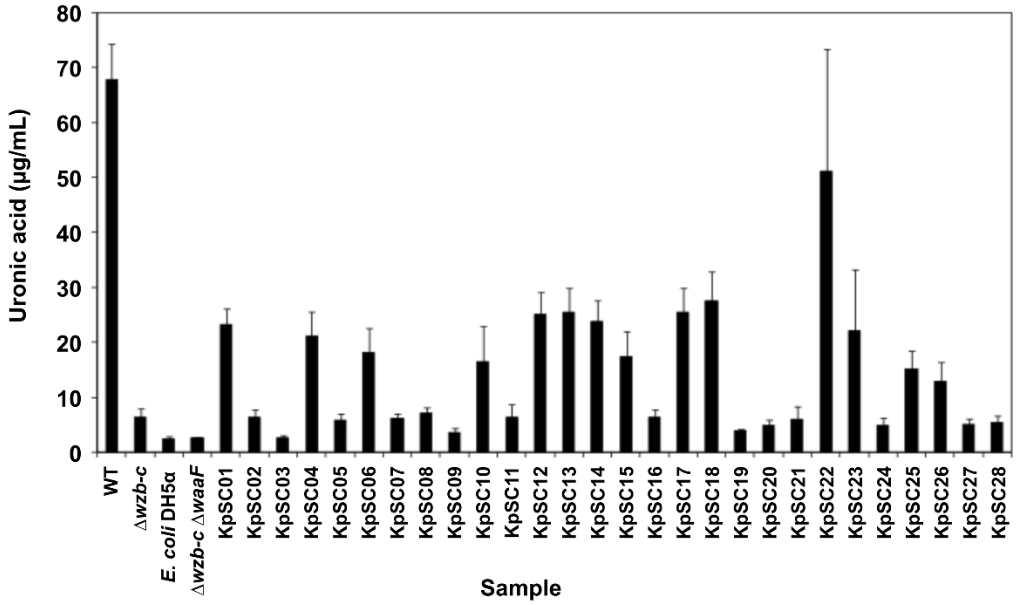

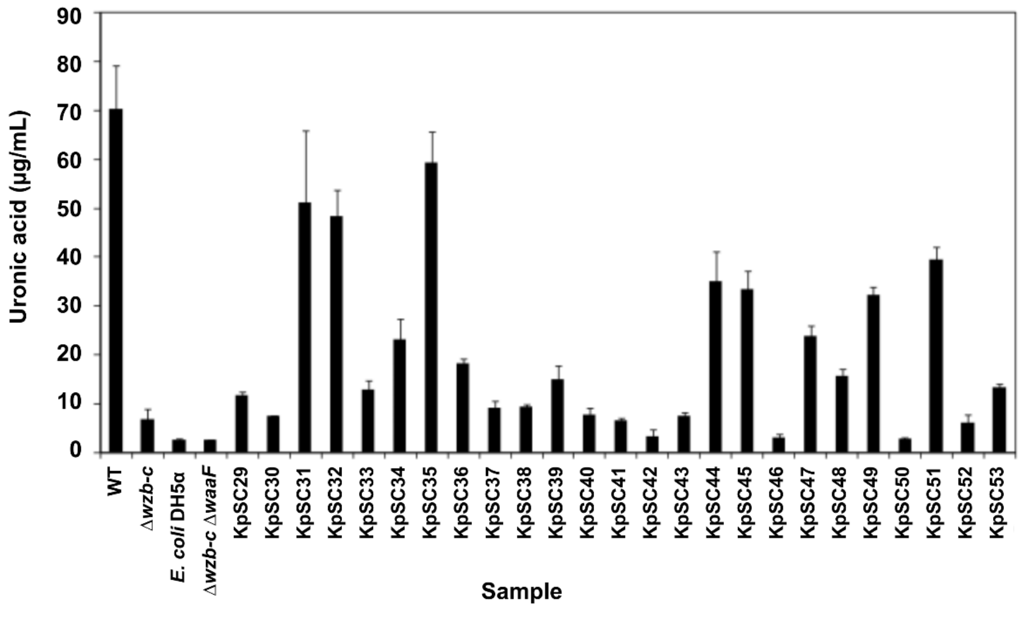

Supplementary Figure 3

3A

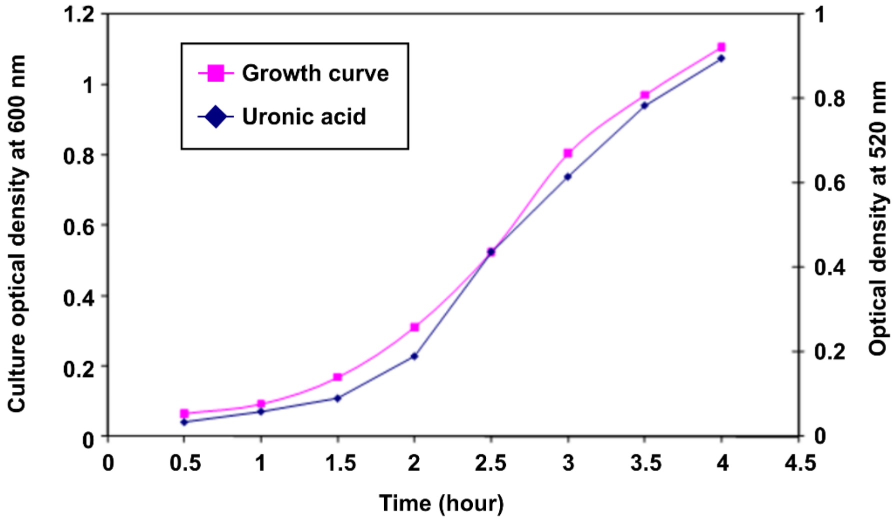

3B

D C B' A B A''

[3)-β-Gal*p*-(1-3)-α-Gal*p*-(1]$_m$-β-Gal*f*-(1-[3)-α-Gal*p*-(1-3)-β-Gal*f*-(1]$_m$-3)-α-Gal*p*-CP

3C

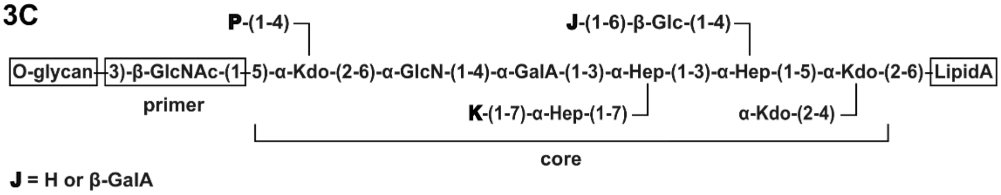

J = H or β-GalA

K = H or β-GalA

P = H or α-Hep

Supplementary Figure 4

Pellet size after centrifugation at 5000×g for 15 minutes

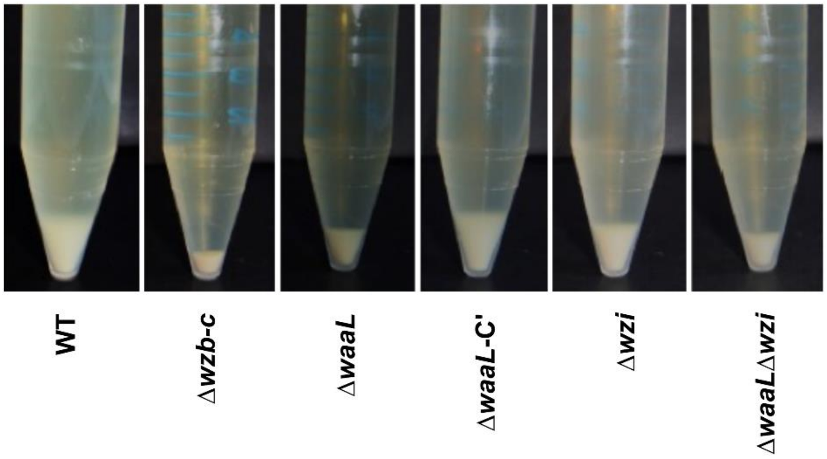

Supplementary Figure 5

A

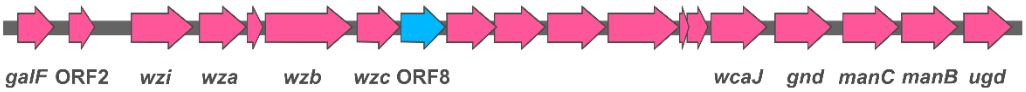

B

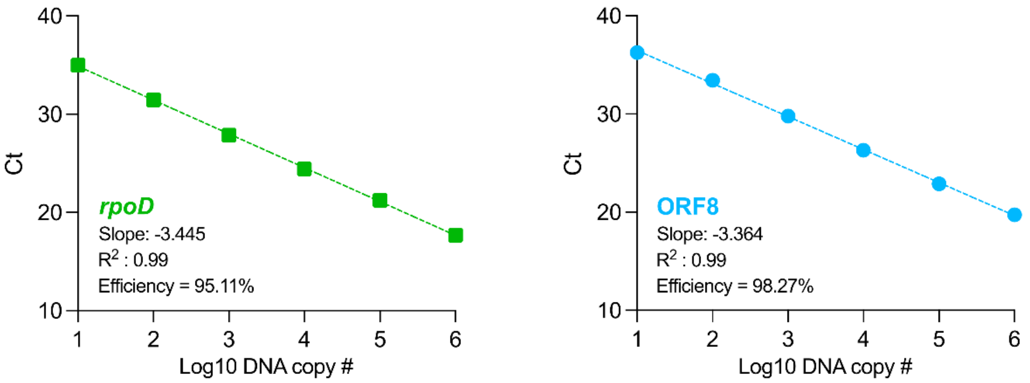

C

B5055 strains	*rpoD*		ORF8	
	Ct (Ave)	DNA copy #	Ct (Ave)	DNA copy #
**WT**	~21	$10^{5.03}$	~22	$10^{5.30}$
**Δ*waaL***	~21	$10^{5.03}$	~22	$10^{5.30}$

SUPPLEMENTARY FIGURES

Supp Figure 1 String test results from *K. pneumoniae* and transposon mutants

The string is measured in cm and the results are the average of three biological samples of the same bacterium. *K. pneumoniae* B5055 (wild type positive control); B5055Δ*wza* (B5055 capsular mutant), B5055Δ*wza*Δ*waaF* (deep core mutant leading to loss of 'O' antigen carbohydrate) and *E. coli* DH5α were used as negative controls; KpSC01-KpSC53 – independent transposon mutants.

Supp Figure 2 Uronate levels in *K. pneumoniae* and mutants

The bacteria were grown in broth to a standardized OD and the bacterial pellet analyzed by a modified carbazole assay for uronic acid. The uronic acid levels were determined using a standard curve using glucuronic acid. Each histogram represents the mean and standard deviation of 6 samples (2 biological replications, 3 technical replicates of each).

Supp Figure 3 Relationship between culture optical density and uronic acid synthesis

(A) An overnight culture of *K. pneumoniae* B5055 was diluted 1/50 in LB-broth and cultured at 37°C, shaking for 4 hours. Samples were removed each 30 minutes and the culture optical density was determined at 600nm. The capsule was prepared from the bacterial pellet (69) and the amount of uronic acid associated with the pellet determined by measuring absorbance at 520nm following addition of dihydroxy-3-phenyl phenol. (B) The LPS O-antigen of O1 *K. pneumoniae* is a polymer of alpha- and beta-linked galactose residues, from (88). (C) The conserved core of *K. pneumoniae* LPS is depicted, from (88).

Supp Figure 4 Pellet size of B5055 and O-antigen mutants

The bacteria were pelleted for analysis of uronic acid. The wild type B5055 produced a large pellet that was reduced in the B5055Δ*wzb-c* and the B5055Δ*waaL* mutants, despite similar colony counts. The pellet size for B5055Δ*wzi* was intermediate. The pellet of B5055Δ*waaL*Δ*wzi* was similar in size to the Δ*waaL* mutant, and the Δ*waaL* mutant pellet *was* restored to wild type levels by complementation (B5055Δ*waaL*-C').

Supp Figure 5 Quantitative PCR of *cps* genes in wild type B5055 and the WaaL mutant

RNA was extracted and stabilised from the wild type B5055 and the defined WaaL mutant. The RNA was analysed by reverse trancriptase quantitative PCR (RTqPCR) to quantify the presence of message for two genes, the constitutive sigma factor rpoD, and ORF8, a putative rhamnosyl transferase present in one of *cps* operons from B5055 (Supp Figure 5A). A Tn5 insertion into ORF8 (KpSc20, Supp Table 2) was negative in the 'string' test and capsule negative (Supp Figures 1B and 2, respectively). The standard curves showing template concentration vs Ct number for the two genes is shown in Supp Figure 5B. The Ct values for the two genes in wild type (WT) and the waaL mutant are shown in Supp Figure 5C. There was no difference in the amount of transcript in the WaaL mutant compared with wild-type, suggesting that the cumulation of the O antigen in the WaaL mutant did not affect transcription of the cps locus.

Supp Table 1 Atomic Force Microscopy measurements of capsule thickness in wild type and capsule mutants

**Strain**	**Capsule thickness measure by AFM (nm) ± Standard Deviation**
B5055	377±48
B5055Δ*wzb-c*	26±6
B5055 WaaL mutant	183±71
B5055 WaaL complemented with *waaL*	316±57

Supp Table 2 Genes with transposon insertions within the cps operon of B5055

**Gene name/ Locus tag**	**Corresponding Protein and it's Function**	**Mutant**	**Tn5 insertion location (bp position)**	**Predicted ORF (bp) and protein (aa) length**
BAA04773.1 / ORF2	putative acid phosphatase	KpSC53	3804[b]	630bp; 209aa
wza	Wza (outer membrane protein), capsule transport	KpSC16, KpSC27, KpSC28	7600[b] 7765[b] Arb PCR 7765[b]	1137bp; 378aa
wzc	Wzc (tyrosine autokinase), Polymerisation and transport of capsule	KpSC11, KpSC24, KpSC38, KpSC40, KpSC41, KpSC43	8693[b] 9241[b] 9461[b] 9157[b] 9743[b] 10186[b]	2169bp; 722aa
BAA04779.1 / ORF8	Glycosyltranferase, Cps synthesis	KpSC20	12438[b]	1122bp; 373aa
wcaJ	Glycosyltranferase, Cps synthesis	KpSC21, KpSC29	20212[b], 19565[b]	1398bp; 465aa
manB	Phosphomannomutase, Cps synthesis	KpSC05	24302[b]	471bp; 157aa
Promoter region	Promoter of cps gene cluster	KpSC37	4753[b]	---

a- *Klebsiella pneumoniae* MGH 78578
b- *Klebsiella pneumoniae* Chedid strain
c- *Klebsiella pneumoniae* 52145
d- pLVPK plasmid

Supp Table 3- Capsule mutants with transposon insertions within the *lps* biosynthesis gene cluster of B5055

**Gene name/ Locus tag**	**Corresponding Protein and it's Function**	**Mutant (Ref)**	**Tn5 insertion location (bp position)**	**Predicted ORF (bp) and protein (aa) length**
wabK	WabK, glucosyl III transferase , outer core lps; Core oligosaccharide biosynthesis	KpSC01, KpSC15, KpSC33	3553[c] 4241[c] 3364[c]	1173bp; 390aa
wabM	WabM, glucosyl II transferase, outer core lps; Core oligosaccharide biosynthesis	KpSC04	5817[c]	993bp; 330aa
waaL	WaaL, lipid A core O-Ag Ligase; Attachment of O-Ag to the core oligosaccharide	KpSC47, KpSC48, KpSC52	5293[c] 5307[c] 4330[c]	1251bp; 416aa
msbB/lpxM	MsbB ; Lipid A biosynthesis lauroyl acyltransferase / LpxM; myristoyl transferase	KpSC31	2596537[a]	975bp; 324aa

Three genes *wabK*, *wabM* and *waaL* of the *waa* operon for core oligosaccharide synthesis showed transposon insertion site. One gene *msbB/lpxM* within the lipid A biosynthesis gene had transposon insertion.

a- *Klebsiella pneumoniae* MGH 78578
b- *Klebsiella pneumoniae* Chedid strain
c- *Klebsiella pneumoniae* 52145
d- pLVPK plasmid

Supp Table 4 Genes with transposon insertion outside the cps and lps biosynthesis gene clusters of B5055

Gene name/ Locus tag	Corresponding Protein and it's Function	Mutant (Ref)	Tn5 insertion location (bp position)	Predicted ORF (bp) and protein (aa) length
galU	Glucose-1-phosphate uridyltransferase/ UDP-glucose pyrophosphorylase (UGPase)	KpSC03, KpSC09, KpSC42	2412983[a], 2412983[a], 2413000[a]	903bp; 300aa
uge	Uridine diphosphate galacturonate 4-epimerase	KpSC19	2720578[a]	1005bp; 334aa
iroC	ABC(ATP Binding Cassette) transporter	KpSC10, KpSC12	94104[d], 94104[d]	3726bp; 1241aa
degP/ htrA	DegP or HtrA or protease Do ; Serine protease, also act a chaperone	KpSC02	10-71 bp AJ430233; Arb PCR	1434bp; 477aa
KPN_01295	Putative Transmembrane efflux protein	KpSC30	1455521[a]	1392bp; 463aa
yfcF	Putative glutathione S-transferase	KpSC14	2963151[a]	639bp; 212aa
cyoA	Cytochrome O ubiquinol oxidase subunit II	KpSC34	435009[a]	909bp; 302aa
pyrC	Dihydroorotase	KpSC49	142463[d]	1347bp; 448aa
*pts*I	PEP- Protein Phosphotransferase system enzyme I	KpSC32	3032906[a]	1728bp; 575aa
ompW	OmpW; Outer membrane protein	KpSC36, KpSC39		
KPN_01648	Putative glyoxylase	KpSC18	1824191[a]	408bp; 135aa
KPN_01649	Hypothetical protein	KpSC17	1823982[a]	1188bp; 395aa
